# Species distribution of nontuberculous mycobacteria identified at a national reference laboratory in Ecuador from 2019 to 2024

**DOI:** 10.3389/fcimb.2026.1965771

**Published:** 2026-10-16

**Authors:** Greta Franco-Sotomayor, Elsy Carvajal, Eva Nicola, Camilo Cardenas Franco, Andrea Belen Salcedo Maldonado, Solon Alberto Orlando, Miguel Angel Garcia-Bereguiain

**Affiliations:** 1 Instituto Nacional de Salud Pública e Investigación, Guayaquil, Ecuador; 2 One Health Research Group, Universidad de Las Américas, Quito, Ecuador; 3 Universidad Católica de Santiago de Guayaquil, Guayaquil, Ecuador; 4 Universidad Ecotec, Samborondón, Ecuador

**Keywords:** Ecuador, line probe assay (LPA), nontuberculous mycobacteria, PRA-hsp65, species identification

## Abstract

Nontuberculous mycobacteria (NTM) are clinically relevant opportunistic pathogens. However, epidemiological data on NTM in low- and middle-income countries like Ecuador remain scarce. This retrospective study characterized the species distribution of NTM identified by molecular methods in the 225 isolates processed at the “Instituto Nacional de Salud Pública e Investigación” in Ecuador from 2019 to 2024. Most isolates were recovered from pulmonary samples (205/225; 91.11%). Their geographic distribution was heterogeneous, with the highest numbers recorded in El Oro, Pichincha, and Los Ríos. However, because all isolates were processed at a single national reference laboratory, this distribution may reflect referral patterns and access to laboratory services. It should not be interpreted as representative of Ecuador’s national NTM epidemiology. Overall, 11 different NTM species were identified, with *M. fortuitum*, *M. intracellulare*, *M. kansasii*, and *M. abscessus* predominating in pulmonary isolates, while *M. fortuitum* and *M. abscessus* predominated in extrapulmonary isolates. The relatively small number of NTM isolates identified over the six-year study period, together with the scarcity of national data, suggests that NTM infections may be underdiagnosed in Ecuador. Strengthening surveillance and diagnostic capacity for NTM in Ecuador is strongly recommended.

## Introduction

Nontuberculous mycobacteria (NTM) are a diverse group of environmental mycobacteria within the family *Mycobacteriaceae*. Although traditionally classified within the genus *Mycobacterium*, this group has been recently reclassified into five genera based on phylogenomic and comparative genomic analyses. NTM encompass more than 200 described species and are widely distributed in natural environments, including soil, dust, air, and both natural and potable water sources (Gupta et al., 2018; Honda et al., 2018; Falkinham, 2021; Maleki and Moaddab, 2025). Their hydrophobic, lipid-rich cell wall favors surface adhesion and confers resistance to disinfectants and antimicrobial agents, enabling persistence in water distribution systems, biofilms, and healthcare environments.

NTM can cause disease in both immunocompromised and immunocompetent individuals, most commonly as pulmonary disease, although extrapulmonary infections involving lymph nodes, skin, and soft tissues are also well documented (Falkinham, 2009; Koh, 2017; Gopalaswamy et al., 2020). In recent years, their rising global incidence, diagnostic challenges, potential misidentification as *Mycobacterium tuberculosis*, and association with nosocomial infections have collectively supported their recognition as emerging pathogens of growing public health concern (Kangabam et al., 2025).

Despite their increasing clinical relevance, the diagnosis of NTM infections remains challenging, especially in low- and middle-income settings. This is mainly due to the nonspecific clinical manifestations of NTM disease, particularly in patients with suspected pulmonary NTM infection, whose symptoms and radiological findings may resemble those of tuberculosis. As a result, NTM infections may be underdiagnosed, misdiagnosed, or diagnosed late, delaying the initiation of appropriate treatment. Therefore, accurate species identification is essential for distinguishing NTM from *Mycobacterium tuberculosis* and guiding appropriate clinical management (CDC, [[NoYear]]; Pennington et al., 2021).

Several NTM diagnostic methods that enable species identification are available. Line-probe assay (LPA) is a molecular method used for NTM identification based on PCR amplification followed by reverse hybridization, allowing differentiation between the *Mycobacterium tuberculosis* complex and several NTM species (Maurya et al., 2017; Dadheech et al., 2023; Xu et al., 2024). The most common LPA assay is the GenoType^®^ Mycobacterium CM/AS assay, commonly used for species-level identification of common and additional NTM species from cultures (Maurya et al., 2017; Dadheech et al., 2023; Xu et al., 2024). Finally, PCR Restriction fragment length polymorphism Analysis (PRA) of the *hsp65* gene involves amplification of the *hsp65* gene, restriction enzyme digestion, and comparison of the resulting fragment patterns with reference databases for NTM species identification (Telenti et al., 1993; da Costa et al., 2010). In addition, Sanger sequencing is widely used for NTM identification through molecular markers such as the *hsp65*, *rpoB*, *and gyrA/gyrB* genes or the 16S–23S ITS region, improving discrimination among closely related species compared to other methods (de Zwaan et al., 2014; Joao et al., 2014; Maboni et al., 2024). More recently, whole-genome sequencing (WGS) has emerged as a high-resolution tool that enables accurate NTM species-level identification, discrimination among closely related species, and assessment of genetic diversity, phylogenetic relationships, and antimicrobial resistance determinants. However, the routine implementation of WGS and even Sanger sequencing remains limited in low- and middle-income countries because of cost, infrastructure, and bioinformatics requirements (Dohál et al., 2021).

In Ecuador, the epidemiological situation of NTM remains poorly characterized, with very limited published data and only a few available studies, suggesting substantial underreporting of NTM infections (Zurita et al., 2014; Ledesma et al., 2022; Echeverria et al., 2023; Orlando et al., 2025). Unlike tuberculosis, which is subject to mandatory epidemiological surveillance and notification in Ecuador, routine reporting of NTM infections remains limited. A recent report highlighted the need to include pulmonary NTM disease as a notifiable condition within national tuberculosis control programs to obtain more accurate prevalence data (Echeverria et al., 2023). Consequently, the lack of systematic reporting and the scarcity of national studies could hinder the estimation of the true burden of NTM infections and contribute to a fragmented understanding of their epidemiology, clinical relevance, and geographic distribution in the country.

This study aimed to provide a first insight into the species distribution of NTM identified at “Instituto Nacional de Salud Pública e Investigación” in Ecuador from 2019 to 2024, describing this distribution across pulmonary and extrapulmonary isolates. This is an isolate-level species distribution study from a national reference laboratory, not a population-based epidemiological study of confirmed NTM disease in Ecuador.

## Methodology

### Study design and samples

This retrospective descriptive study included all nontuberculous mycobacterial (NTM) isolates identified by the “Instituto Nacional de Salud Pública e Investigación” (INSPI), Ecuador, between 2019 and 2024. A total of 249 isolates were initially processed during the study period. Of these, 24 isolates were excluded because conclusive species-level identification could not be reached. After applying this exclusion criterion, 225 NTM isolates were included in the study. Eligible isolates originated from patients with clinical suspicion of mycobacterial infection, defined by a positive acid-fast bacilli (AFB) smear, clinical presentation consistent with pulmonary or extrapulmonary mycobacterial infection, and a negative GeneXpert MTB/RIF result.

Isolates were classified by anatomical origin as pulmonary, including sputum, bronchoalveolar lavage, bronchial lavage, bronchial aspirates, and gastric aspirates, or extrapulmonary, including urine, abscess, stool, lymph node, tissue or biopsy, cerebrospinal fluid, pleural fluid, drainage fluid, and surgical wound samples. Demographic and laboratory variables included year of diagnosis, patient sex and age, sample type, geographic origin, NTM species identified, and the molecular identification method used.

Detailed individual clinical records, including symptoms, imaging findings, and microbiological confirmation, were not accessible for this retrospective, laboratory-based study. However, all isolates included had met the general clinical suspicion criteria described above, as applied by the INSPI diagnostic algorithm, even though patient-level clinical documentation was not available to the study team. Moreover, the clinical, radiological, and microbiological criteria required to establish confirmed NTM disease according to ATS/ERS/ESCMID/IDSA guidelines (Daley et al., 2020) were not available in the laboratory database. Therefore, the included records were analyzed as NTM isolates rather than confirmed cases of NTM disease.

### Nontuberculous mycobacteria culture and heat inactivation

NTM isolates were cultured using the Kudoh–Ogawa method, as previously described by our laboratory (Franco-Sotomayor et al., 2020; Castro-Rodriguez et al., 2024). Mycobacterial colonies were harvested from cultures, resuspended in Tris-EDTA (TE) buffer (10 mM Tris–HCl, 1 mM EDTA, pH 8.0), and inactivated at 95 °C for 15 min. After heat inactivation, all isolates were centrifuged for 5 min at 10,000 x * g*, and the supernatant was directly used for DNA isolation as described below for each diagnosis method.

### Line-probe assay for NTM species identification

Of the 225 isolates, 133 were identified using the GenoType Mycobacterium CM assay version 2.0 (Hain Lifescience, Nehren, Germany), a DNA strip line probe assay for differentiating the *M. tuberculosis* complex from common NTM species. Genomic DNA was extracted from the heat-inactivated culture supernatant described above using the GenoLyse DNA Extraction kit (Hain Life Science), followed by PCR amplification with biotinylated primers according to the manufacturer’s instructions. The resulting amplicons were denatured, hybridized to membrane strips containing species-specific oligonucleotide probes, washed under stringent conditions, and detected using a streptavidin alkaline phosphatase conjugate with chromogenic substrate. Species identification was assigned by comparing the banding pattern with the manufacturer’s interpretation chart. Each run included internal strip controls, as well as positive and negative controls (Maurya et al., 2017; Xu et al., 2024).

### PCR–restriction fragment length polymorphism analysis of the hsp65 gene

The remaining 92 isolates were identified by PRA-*hsp65* following the method published elsewhere (Telenti et al., 1993; Hernández-Toloza et al., 2016). Briefly, following DNA isolation with QIAamp DNA Mini Kit (QIAGEN), a 441-bp fragment of the *hsp65* gene was amplified from extracted DNA with the primers TB11 (5′-ACCAACGATGGTGTGTCCAT-3′) and TB12 (5′-CTTGTCGAACCGCATACCCT-3′), using 45 cycles of amplification (94 °C for 1 min, 60 °C for 1 min, and 72 °C for 1 min), followed by a final extension at 72 °C for 10 min (Telenti et al., 1993). Each PCR run included a positive control, consisting of a *Mycobacterium abscessus* isolate previously characterized by WGS and MALDI-TOF mass spectrometry, and a negative control consisting of the same Tris-EDTA buffer used for heat inactivation of cultures prior to DNA extraction. The amplicon was digested separately with the restriction endonucleases BstEII and HaeIII, and the resulting fragments were resolved by electrophoresis on a 3% agarose gel. Species were identified by comparing the restriction-fragment-length patterns with the publicly available PRASITE algorithm and previously published reference patterns (Telenti et al., 1993; Chimara et al., 2008; Hernández-Toloza et al., 2016).

### Statistical analysis

For each of the 11 NTM species, a separate 2 × 2 contingency table was constructed comparing the presence of that species versus all other species between pulmonary and extrapulmonary isolates. Fisher’s exact test was applied to each table. To account for multiple comparisons, the resulting p-values were adjusted using the Benjamini–Hochberg false discovery rate (FDR) method, with FDR-adjusted p-values < 0.05 considered statistically significant. All analyses were performed in R version 4.3.1 (R Foundation for Statistical Computing, Vienna, Austria).

## Results

### Geographic distribution of NTM

NTM isolates were heterogeneously distributed across the study locations. El Oro had the highest number of isolates (48/225; 21.33%), followed by Pichincha (40/225; 17.78%) and Los Ríos (35/225; 15.56%). Together, these three provinces accounted for 123 isolates, representing 54.67% of the total (**Figure 1**). The remaining isolates were distributed across additional provinces.

**Figure 1:**
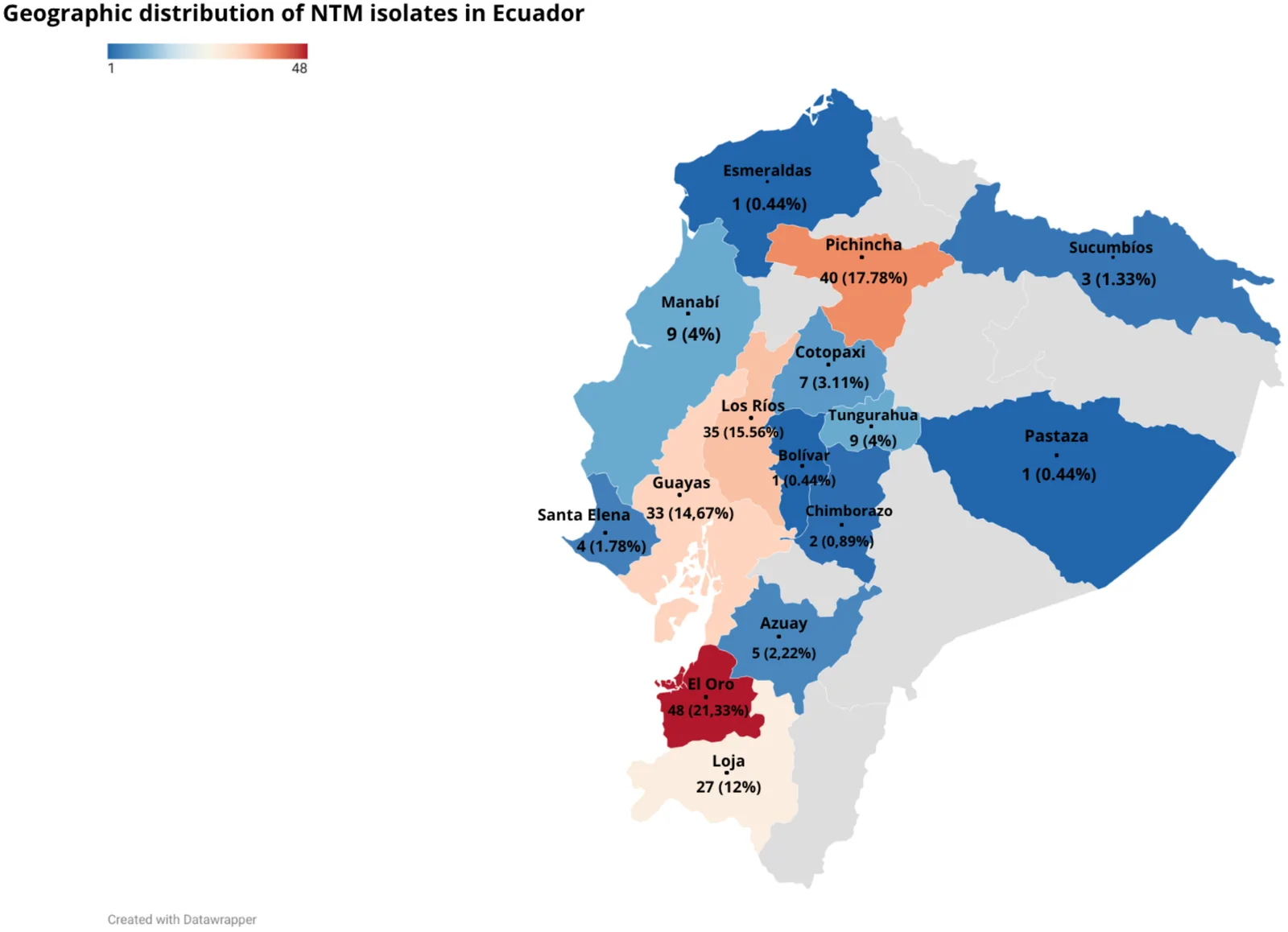
Geographic distribution of NTM isolates in Ecuador by province. Choropleth shading indicates the number of isolates per province; labels give the number of isolates and the percentage of the national total (n = 225). Provinces without recorded isolates are unshaded.

### General characterization of NTM isolates

During the study period from 2019 to 2024, a total of 225 NTM isolates were identified, of which 205 were classified as pulmonary isolates and 20 as extrapulmonary isolates according to the anatomical origin of the corresponding clinical samples. The isolates were obtained from 130 male and 95 female patients, whose ages ranged from 1 to 92 years. The highest number of isolates was recorded in 2021 (n = 52), followed by 2022 (n = 43) (**Figure 2**). Regarding the molecular identification methods, PRA-hsp65 was predominantly used in the early years of the study (2019–2020), while LPA was implemented from 2022 onward; both methods were used concurrently in 2021 but were applied to different subsets of isolates (no single isolate was analyzed by both methods simultaneously) (**Table 1**; **Figure 2**).

**Figure 2:**
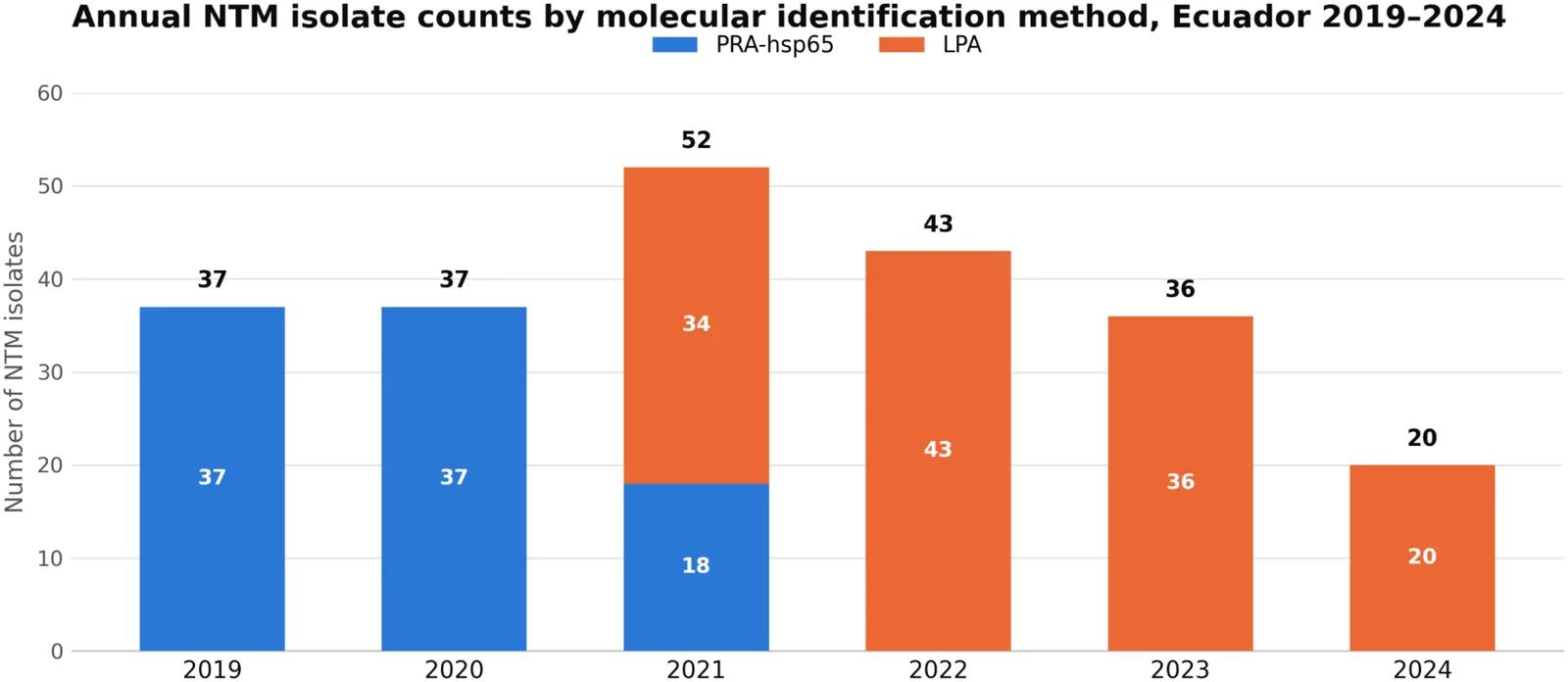
Annual NTM isolate counts by molecular identification method, Ecuador 2019–2024. Bars show the number of isolates identified by PRA-hsp65 (blue) and LPA (orange) each year; both methods were used concurrently only in 2021, on separate, non-overlapping subsets of isolates. Numbers above each bar indicate the total annual isolate count (n = 225).

**Table 1:** Annual distribution of NTM isolates according to sex ratio, age range, and molecular identification method, 2019–2024.

Year	Number of NTM isolates	Sex ratio (M/F)	Age range, years	Method used
2019	37	1.06	12-89	PRA
2020	37	1.64	5-89	PRA
2021	52	1.60	10-92	PRA & LPA*
2022	43	1.39	10-88	LPA
2023	36	1.57	2-88	LPA
2024	20	0.82	1-85	LPA

M/F sex ratio, number of male patients/number of female patients.

*In 2021, PRA-hsp65 and LPA were applied to different, non-overlapping subsets of isolates.

Overall, 11 NTM species were identified: *M. avium* and *M. intracellulare* (members of the *M. avium* complex), *M. abscessus*, *M. fortuitum*, *M. chelonae*, *M. kansasii*, *M. szulgai*, *M. gordonae*, *M. flavescens*, *M. terrae*, and *M. scrofulaceum* (**Table 2**). The annual proportional distribution of the predominant species is shown in **Figure 3**.

**Table 2:** NTM species distribution across pulmonary and extrapulmonary isolates included in this study.

Species	Anatomical specimen group				
	Pulmonary (n=205)		Extrapulmonary (n=20)		FDR-adjusted p-value
	n	%	n	%	
*M. avium (M. avium complex)*	14	6.83%	1	5.00%	1.00
*M. intracellulare (M. avium complex)*	37	18.05%	2	10.00%	1.00
*M. abscessus*	28	13.66%	5	25.00%	1.00
*M. fortuitum*	40	19.51%	6	30.00%	1.00
*M. chelonae*	12	5.85%	0	0.00%	1.00
*M. kansasii*	33	16.10%	2	10.00%	1.00
*M. szulgai*	2	0.98%	0	0.00%	1.00
*M. gordonae*	7	3.41%	0	0.00%	1.00
*M. flavescens*	13	6.34%	2	10.00%	1.00
*M. terrae*	10	4.88%	2	10.00%	1.00
*M. scrofulaceum*	9	4.39%	0	0.00%	1.00

Percentages were calculated within each anatomical group: pulmonary isolates (n = 205) and extrapulmonary isolates (n = 20).

**Figure 3:**
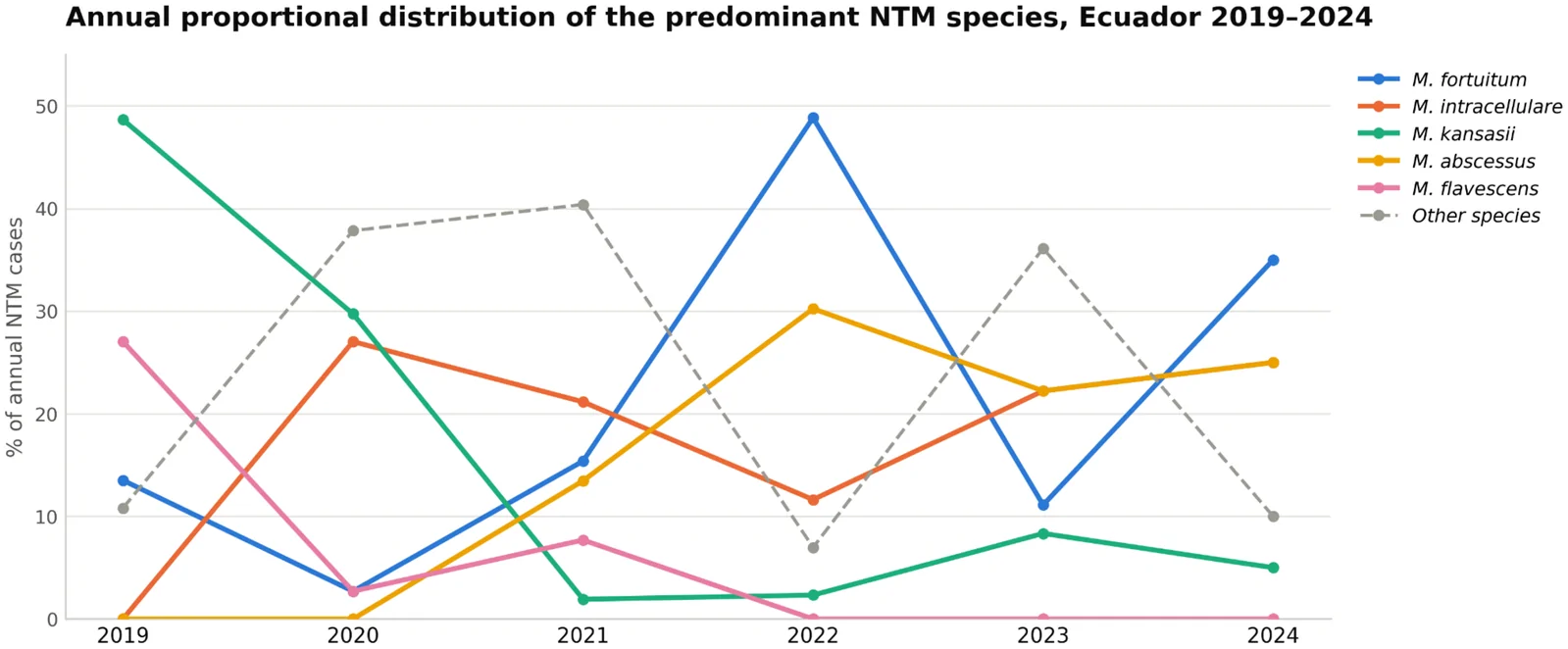
Annual proportional distribution of the predominant NTM species, Ecuador 2019–2024. Lines show the percentage of annual NTM isolates (n = 225) identified as each of the five most frequently detected species (*M. fortuitum*, *M. intracellulare*, *M. kansasii*, *M. abscessus*, and *M. flavescens*); the remaining six species (*M. avium*, *M. chelonae*, *M. terrae*, *M. scrofulaceum*, *M. gordonae*, and *M. szulgai*) are grouped as “Other species.”.

### Sex and age distribution of NTM

Overall, 130 isolates (57.78%) were recovered from male patients and 95 (42.22%) from female patients, resulting in a male-to-female ratio of 1.37 (**Table 1**). The male predominance was confined to the pulmonary isolate group (≈60% male), whereas the smaller extrapulmonary isolate group (n = 20) showed a female predominance of approximately 3:1. The sex balance also varied geographically: in El Oro, the province with the most isolates (48/225), the distribution was nearly balanced, with a slight female predominance (23 male vs. 25 female; ratio 0.92), in contrast to Los Ríos and Pichincha, where male patients clearly predominated (ratio 2.18 and 1.86, respectively).

### NTM species distribution among pulmonary and extrapulmonary isolates

Most NTM isolates were pulmonary (205/225; 91.11%), while a smaller proportion were extrapulmonary (20/225; 8.89%). A detailed breakdown of isolates by sample type is provided in **Supplementary Table 1**. Among pulmonary isolates, the most frequently identified species were *M. fortuitum* (40/205; 19.51%), *M. intracellulare* (37/205; 18.05%), *M. kansasii* (33/205; 16.10%), and *M. abscessus* (28/205; 13.66%). Among extrapulmonary isolates, *M. fortuitum* and *M. abscessus* were the most frequently identified species, accounting for 30% (6/20) and 25% (5/20) of isolates, respectively. Fisher’s exact test with FDR correction did not reveal statistically significant differences in species distribution between pulmonary and extrapulmonary isolates (all FDR-adjusted *p*-values > 0.05; **Table 2**).

## Discussion

This study describes the distribution of NTM species identified by molecular methods among 225 isolates processed at a single national reference laboratory at “Instituto Nacional de Salud Pública e Investigación” of Ecuador between 2019 and 2024. It is an isolate-level study rather than a population-based epidemiological investigation of confirmed NTM disease, but it nevertheless contributes to the limited information available on NTM in Ecuador. In total, 11 different NTM species were found, including *M. avium* and *M. intracellulare* (*M. avium* complex), *M. abscessus*, *M. fortuitum*, *M. chelonae*, *M. kansasii*, *M. szulgai*, *M. gordonae*, *M. flavescens*, *M. terrae*, and *M. scrofulaceum*.

This study also highlights the heterogeneous geographic distribution of NTM isolates and the clear predominance of isolates recovered from pulmonary samples. More importantly, the relatively small number of isolates reported at Ecuador’s national reference laboratory over six years, in a country of approximately 17 million inhabitants, suggests possible underdiagnosis of NTM infections. In this context, the geographic concentration of isolates in El Oro, Pichincha, and Los Ríos may partly reflect differences in laboratory capacity, referral patterns, and surveillance infrastructure rather than true regional variation in NTM burden.

The male predominance observed in our study was mainly found among patients from whom pulmonary isolates were recovered. This finding is broadly consistent with reports of pulmonary NTM disease, which have described a higher frequency among men, potentially associated with the higher prevalence of predisposing factors such as chronic obstructive pulmonary disease (COPD) and smoking history (Glassroth, 2008). In contrast, the female predominance observed among patients with extrapulmonary isolates in our study is consistent with population-based surveillance data from other countries, such as the United States, where skin and soft-tissue NTM infections were more frequently reported among women, whereas disseminated infections occurred predominantly among men (Henkle et al., 2017). The underlying reasons for sex-related differences in extrapulmonary NTM infections remain unclear and deserve further research. However, differences in host immunity or host–pathogen interaction have been proposed as more likely explanations than differences in environmental exposure (Mora et al., 2021). Notably, 2024 was the only year in which women outnumbered men in overall NTM isolates, though the small number of isolates that year (n = 20) limits interpretation of this observation. Regarding age distribution, patients spanned all age groups, from 1 to 92 years, and a similarly broad age range was observed throughout the study period. The overall median age was 54 years, indicating that NTM were identified predominantly, but not exclusively, in middle-aged and older adults, as reported elsewhere (Glassroth, 2008; Henkle et al., 2017).

Most isolates in our study were recovered from pulmonary samples (91.11%), consistent with the predominance of respiratory isolates and pulmonary presentations reported in the global NTM literature (Prevots et al., 2023). Among pulmonary isolates, *M. fortuitum*, *M. intracellulare*, *M. kansasii*, and *M. abscessus* were the most frequently identified species. This distribution partially aligns with reports from Latin America, where *M. avium* complex, *M. abscessus*, and *M. fortuitum* have been described as predominant, although *M. kansasii* and *M. intracellulare* were more prominent in our study (Mora et al., 2021). In extrapulmonary isolates, *M. fortuitum* and *M. abscessus* predominated, consistent with the recognized role of rapidly growing mycobacteria as causative agents of skin, soft tissue, and lymph node infections, as documented in a recent case series from neighboring Colombia, where *M. fortuitum* complex (46%) and *M. abscessus* complex (31%) were the predominant extrapulmonary isolates (Parra-Villamil et al., 2025). No statistically significant differences in species distribution were detected between pulmonary and extrapulmonary isolates; however, this result should be interpreted cautiously given the small number of extrapulmonary isolates (n = 20), which limited the statistical power of this comparison. Larger prospective studies are therefore needed to better characterize the clinical spectrum of NTM disease in Ecuador.

This study has several limitations. First, all isolates were processed at a single national reference laboratory. Therefore, their geographic distribution may reflect differences in referral patterns and access to laboratory services rather than the true geographic distribution of NTM infections in Ecuador. Consequently, these findings should not be interpreted as representative of the national epidemiology of NTM. Second, the small number of extrapulmonary isolates (n = 20) limited the statistical power to detect differences in species distribution between pulmonary and extrapulmonary isolates. Therefore, the absence of statistically significant differences should not be interpreted as evidence that the species distributions were equivalent between these anatomical groups. Third, PRA-hsp65 and LPA were applied to mutually exclusive sets of isolates; no isolate was analyzed using both methods. PRA-hsp65 was predominantly used during 2019–2020, whereas LPA was used from 2022 onward. In 2021, both methods were used, but each was applied to a separate, non-overlapping subset of isolates. As a result, no valid comparison of diagnostic performance between these two methods can be drawn from this dataset, since any apparent differences in species distribution may equally reflect temporal changes in circulating NTM species rather than true differences between methods; parallel testing of stored isolates with both methods was not possible. The choice of method used at “Instituto Nacional de Salud Pública e Investigación” each year depended on the budget available from the Ministry of Public Health, since PRA-hsp65 is a lower-cost in-house method compared with LPA, which relies on a commercial supplier. Finally, the scarcity of prior national data on NTM in Ecuador limited our ability to contextualize these findings against historical trends. Another limitation is the inherently limited resolution of the molecular methods used. Both PRA-hsp65 and LPA have lower discriminatory power than sequencing-based approaches (Daley et al., 2020), particularly for closely related taxa such as the *M. abscessus* complex, *M. fortuitum* and related species, the *M. terrae* complex, and certain *M. kansasii* variants, where band patterns may overlap or intraspecies polymorphisms may go undetected (Telenti et al., 1993; Chimara et al., 2008; Hernández-Toloza et al., 2016). Sequencing-based methods (e.g., hsp65, rpoB, or ITS gene sequencing) would provide higher-resolution species-level discrimination in these groups.

In conclusion, this is the first longitudinal overview of the diversity of NTM isolated at the “Instituto Nacional de Salud Pública e Investigación” in Ecuador over a six-year period, showing 11 NTM species. Most isolates were recovered from pulmonary samples and were concentrated among patients in a small number of densely populated provinces. *M. fortuitum*, *M. intracellulare*, *M. kansasii*, and *M. abscessus* were the most frequently identified species. Because these data were obtained from a single national reference laboratory, they should not be considered fully representative of NTM epidemiology across Ecuador. Nevertheless, the identification of only 225 NTM isolates over six years, together with the limited availability of national epidemiological data, highlights the significant limitations in surveillance and diagnostic capacity in the country. Strengthening laboratory capacity and epidemiological surveillance, as well as incorporating complementary species-identification methods such as Sanger sequencing, is therefore recommended. These measures would contribute to a more accurate assessment of the burden and distribution of NTM infections and support appropriate clinical management in Ecuador.

## Data Availability

The original contributions presented in the study are included in the article/**Supplementary Material**. Further inquiries can be directed to the corresponding author.

## Supplementary material

Supplementary Table 1 Distribution of NTM isolates by sample type (n = 225).
