# Supplementary material for: Species distribution of nontuberculous mycobacteria identified at a national reference laboratory in Ecuador from 2019 to 2024
Source: Front Cell Infect Microbiol. 2026 Oct 16;16:1965771. doi: 10.3389/fcimb.2026.1965771 (PMC13719316; doi:10.3389/fcimb.2026.1965771)
Supplement: Supplementary file 1 [file Table1.docx]

**Supplementary Table 1. Distribution of NTM isolates by sample type (n = 225).**

| **Clinical group** | **Sample type** | **n** | **%** |
| --- | --- | --- | --- |
| **Pulmonary** | Sputum | 191 | 84.89% |
|  | Bronchial aspirate | 6 | 2.67% |
|  | Bronchoalveolar lavage (BAL) | 4 | 1.78% |
|  | Bronchial lavage | 1 | 0.44% |
|  | Gastric aspirate | 3 | 1.33% |
| **Extrapulmonary** | Urine | 6 | 2.67% |
|  | Abscess | 3 | 1.33% |
|  | Stool | 3 | 1.33% |
|  | Lymph node | 2 | 0.89% |
|  | Tissue/biopsy | 2 | 0.89% |
|  | Cerebrospinal fluid (CSF) | 1 | 0.44% |
|  | Pleural fluid | 1 | 0.44% |
|  | Drainage fluid | 1 | 0.44% |
|  | Surgical wound | 1 | 0.44% |
| **Total** |  | 225 | 100% |

**Note:** *n* indicates the number of isolates recovered from each sample type. Percentages were calculated relative to the total number of isolates included in the study (*n* = 225).
